# *Capoeta coadi*, a new species of cyprinid fish from the Karun River drainage, Iran based on morphological and molecular evidences (Teleostei, Cyprinidae)

**DOI:** 10.3897/zookeys.572.7377

**Published:** 2016-03-16

**Authors:** Nisreen H. Alwan, Halimeh Zareian, Hamid Reza Esmaeili

**Affiliations:** 1 Senckenberg Research Institute and Museum of Nature, Frankfurt 60325, Germany; 2 Modern University for Business and Science, School of Health Sciences, P.O. Box 113-7501, Beirut, Lebanon; 3 Ichthyology and Molecular Systematics Laboratory, Department of Biology, College of Sciences, Shiraz 71454, Iran

**Keywords:** *Capoeta damascina* species complex, COI, *Cyt b*, Persian Gulf, phylogenetic relationships

## Abstract

As presently recognized, the genus *Capoeta* includes 24 species, nine of which are known to occur in Iran (*Capoeta aculeata*, *Capoeta capoeta*, *Capoeta buhsei*, *Capoeta damascina*, *Capoeta fusca*, *Capoeta heratensis*, *Capoeta mandica*, *Capoeta saadii* and *Capoeta trutta*) and are distributed in almost all Iranian basins except Sistan and Mashkid. *Capoeta coadi* **sp. n.** is a new species from the Karun River, southern Iran, draining into the Arvand Rud (Shatt al-Arab) which drains into the Persian Gulf. It is distinguished from all other species of *Capoeta* by the combination of the following characters: elongate and usually cylindrical body; 8–9 branched dorsal-fin rays; last unbranched dorsal-fin ray weakly to moderately ossified and serrated along 1/3–2/3 of its length; scales small; 70-84 in lateral line (total); 12–17 scales between dorsal-fin origin and lateral line; 9-11 scales between anal-fin origin and lateral line; 26–32 circum-peduncular scales; 10–13 gill rakers on lower limb of first gill arch; 45–47 total vertebrae; one posterior pair of barbels; bright golden-greenish or silvery body coloration in life; length of the longest dorsal-fin ray 15–22% SL; head length 23–26% SL; mouth width 7–10% SL. *Capoeta coadi* is also distinguished from all other congeners in the Iranian drainages by fixed diagnostic nucleotide substitutions in the mtDNA COI barcode region and *cyt b*. It is nested in the *Capoeta damascina* species complex.

## Introduction

The Middle East is a transition zone between three major biogeographic units, the Palaearctic, the Afrotropical, and the Oriental realms. It served as an important crossroad of biotic exchange resulting in an outstanding biological diversity of freshwater fishes (Durand et al. 2002, Krupp et al. 2009). Lying between major drainages of the Nile in Africa to the west, the Indus in southern Asia to the east and the Caspian and Black Sea drainages to the north, the Tigris-Euphrates River drainage is the largest river system in the Middle East and has high fish diversity, especially in cyprinid fishes.

*Capoeta* Valenciennes in Cuvier and Valenciennes 1842 is an example of a cyprinid genus widely spread in the Middle East (Krupp and Schneider 1989). Being found in a wide range of habitats, species of this genus display considerable morphological variability (e.g., scale counts and colour pattern) and the extent of morphological plasticity and genetic variability remain to be determined. As a consequence, there has been considerable disagreement regarding the status of several species. However, *Capoeta* is considered monophyletic (Krupp 1985, Küçük et al. 2009).

Members of the genus *Capoeta* are cyprinids characterized by having an elongate, cylindrical body and a short dorsal fin. They have three to five unbranched and 5–9 branched dorsal-fin rays, the last unbranched ray being ossified and serrated. All species have three unbranched and 5 branched anal-fin rays. Scales are usually small. Mouth is inferior and the lower lip is covered with a horny sheath. One pair of barbels (rarely two) is present and the pharyngeal teeth are arranged in three rows. The shape of the mouth as well as the pharyngeal teeth are nearly identical in all species, which indicate their adaptation to the same mode of feeding. This combination of character states distinguishes *Capoeta* from all other cyprinids (Krupp 1985, Krupp and Schneider 1989).

As presently recognized, the genus *Capoeta* includes about 24 species (Eschmeyer and Fricke 2016) in different phylogenetic groups widely distributed in many river drainages and basins in southwestern Asia except the Arabian Peninsula (Alwan 2011, Levin et al. 2012). Levin et al. (2012) studied the phylogenetic relationships of the genus *Capoeta* based on complete mitochondrial gene for cytochrome *b* sequences obtained from 20 species from the overall range of the genus. Three main groups were detected: the Mesopotamian group (*Capoeta trutta* group), the Anatolian-Iranian group (*Capoeta damascina* group) and the Aralo-Caspian group (*Capoeta capoeta* group).

Members of the *Capoeta damascina* species group, characterized by having small scales, include *Capoeta buhsei* Kessler, 1877, *Capoeta caelestis* Schöter, Özuluğ & Freyhof, 2009, *Capoeta damascina* (Valenciennes, 1842), *Capoeta kosswigi* Karaman, 1969, *Capoeta saadii* (Heckel, 1847), and *Capoeta umbla* (Heckel, 1843) (Alwan 2011). Based on phylogenetic analyses of cytochrome c oxidase subunit I (COI) and the large subunit (LSU or 28S) ribosomal RNA gene sequences Alwan (2011) identified two main lineages within what we will refer to in this paper, as the “*Capoeta damascina* species complex”. A western lineage is represented by *Capoeta caelestis*, *Capoeta damascina* and *Capoeta umbla* and an eastern lineage represented by *Capoeta buhsei*, *Capoeta saadii*, and a new undescribed species.

Traditionally, *Capoeta damascina* is recorded from Tigris, Mond, Kor, Esfahan, Dasht-e Kavir, Namak Lake, Kor River, Lake Maharlu, Persian Gulf (now Persis), Kerman-Na’in, Dasht-e Lut, Sirjan, Hormuz, and Hamun-e Jaz Murian basins in Iran (Nikol’skii 1899, Berg 1949, Kähsbauer 1964, Armantrout 1980, Rainboth 1981, Bianco and Banarescu 1982, Ghorbani Chafi 2000, Jalali et al. 2005, Esmaeili et al. 2010, Bahrami Kamangar et al. 2012). Its distribution over such wide range of isolated water bodies, raises questions regarding the status of *Capoeta damascina*. Currently, *Capoeta damascina* s.l. represents a complex of closely related species with high intraspecific and comparatively low interspecific variation (Alwan 2011, Levin et al. 2012). Now, three species of *Capoeta* from Iranian water bodies are recognized as being members of *Capoeta damascina* species complex group: *Capoeta buhsei*, *Capoeta saadii* (Iranian populations were considered as *Capoeta damascina*) (see Alwan 2011, Levin et al. 2012), and a new undescribed species from the Karun (Karoun) River drainage. It is described here as a new species, *Capoeta coadi*.

## Material and methods

After anaesthesia, fishes were either fixed in 5% formaldehyde, and stored in 70% ethanol, or directly fixed in 99% ethanol (for molecular studies). Measurements were made with a digital caliper and recorded to 0.01 mm. All measurements were made point to point, and never by projections. Methods for counts and measurements follow Hubbs and Lagler (1958) and Krupp (1983). Standard length (SL) was measured from the tip of the snout to the end of the hypural complex. The length of the caudal peduncle was measured from behind the base of the last anal-fin ray to the end of the hypural complex. The last two branched rays articulating on a single pterygiophore in the dorsal and anal fins are counted as “1½”.The holotype is included in the calculation of means and SD.

Abbreviations used: SL, standard length; HL, lateral head length.

Abbreviations used for museum collections: Zoological Museum of Shiraz University, Collection of Biology Department, Shiraz, Iran (ZM-CBSU), the Senckenberg Research Institute and Natural History Museum (SMF: Frankfurt, Germany), and the private collection of Jörg Freyhof (FSJF: Fischsammlung J. Freyhof).

### DNA extraction and PCR amplification protocol

For DNA sequencing, specimens were directly fixed in 99% molecular grade ethanol. Mitochondrial DNA was extracted using Salt method (Bruford et al. 1992). The standard vertebrate DNA barcode region of the COI (cytochrome c oxidase subunit 1) and cytochrome *b* (*cyt b*) were amplified using primer pairs named FishF1-5'TCAACCAACCACAAAGACATTGGCAC3’ and FishR1-5'TAGACTTCTGGGTGGCCAAAGAATCA3’ (Ward et al. 2005) and L14724-5'GTGACTTGAAAAACCACCGTTG3’ and H15915-5'CAACGATCTCCGGTTTAGAAGAC3’ (Xiao et al. 2001) or GluF- 5'AACCACCGTTGTATTCAACTACAA3’ and H-15560 5`TAGGCRAATAGGAAR TATCA3` (Machordom and Doadrio 2001), respectively.

Purification and sequencing of the PCR products were conducted at Macrogen Korea Laboratories using the aforementioned primer sets.

### Molecular data analyses

Data processing and sequence assembly was done in BioEdit 7.2.5 (Hall 1999); MEGA6 (Tamura et al. 2013) was used to create a DNA sequence alignment. No indications of unexpected stop-codons or nuclear copies of mitochondrial fragments occurred in any sequences. All generated DNA barcodes and *cyt b* were deposited in the NCBI GenBank. The most appropriate sequence evolution model for the given data was determined with Modeltest (Posada and Crandall 1998) as implemented in the MEGA6 software, treating gaps and missing data with the partial deletion option under 95% site coverage cut-off. The model with the lowest BIC (Bayesian Information Criterion) score is considered the best model to describe the substitution pattern for each gene. To explore species phylogenetic relationships, trees were generated using Maximum Likelihood analysis with 10,000 bootstrap replicates in RaxML 7.2.5 (Stamatakis 2006) under the GTR+G model of nucleotide substitution, with fast bootstrap and also Bayesian analysis (BA), using the Markov Chain Monte Carlo method (MCMC), with 6,000,000 generations under the most generalizing model (GTR+G+I) using Mr. Bayes 3.1.1 (Huelsenbeck and Ronquist 2001). Screening for diagnostic nucleotide substitutions was performed manually from the sequence alignment. As an appropriate outgroup to root the constructed phylogenetic hypothesis, we included the distantly related *Cyprinus carpio*.

## Results

### Morphological assessments

#### Capoeta coadi sp. n.

Taxon classificationAnimaliaCypriniformesCyprinidae

http://zoobank.org/4B5B0984-0C65-4B6D-97CC-31245E179D13

Figs 1 , 2 , 3

##### Holotype.

ZM-CBSU Z190, 157 mm SL; Iran, Kohgiluyeh and Boyer Ahmad prov., Beshar (Bashar) River at Tale Gah village, Karun River drainage, 30°47'27"N, 51°25'13"E.

##### Paratypes.

ZM-CBSU Z191, 6, 91–157 mm SL; same data as holotype. ZM-CBSU J520, 1, 107 mm SL; ZM-CBSU Z275, 12, 105–152 mm SL; Iran, Kohgiluyeh and Boyer Ahmad prov., Beshar (Bashar) River at Tale Gah village, Karun River drainage, 30°47'27"N, 51°25'13"E. 15 December 2014, G. Sayyadzadeh, R. Khaefi, A. Khajehpanah. ZM-CBSU J526, 1, 98 mm SL; ZM-CBSU J533, 1, 114 mm SL; ZM-CBSU J535, 1, 97 mm SL; ZM-CBSU J540, 1, 67 mm SL; All from Iran, Kohgiluyeh and Boyer Ahmad prov., Beshar River at Tange sorkh, Karun River drainage, 30°26'14"N, 51°45'48"E. 24 July 2011, R. Zamaneian Nejad, S. Mirgheiasi, S. Ghasemian. ZM-CBSU J444, 2, 73–90 mm SL; ZM-CBSU J447, 2, 76–111 mm SL; ZM-CBSU J450, 1, 86 mm SL; ZM-CBSU J452, 1, 107 mm SL; ZM-CBSU J459, 2, 104–120 mm SL; ZM-CBSU J464, 1, 110 mm SL; all from Iran, Kohgiluyeh and Boyer Ahmad prov., Beshar River at Mokhtar village, Karun River drainage, 30°40'31"N, 51°31'26"E. 25 May 2011, R. Zamaneian Nejad.

##### Additional material.

ZM-CBSU 7880–7881, 2, 96.69–158.12 mm SL; Iran, Fars prov., Sepidan city, Gorgu River, a tributary of Beshar River, north of Sepidan city, Karun River drainage, 30°21.283'N, 51°45.754'E. 2006. H.R. Esmaeili, A. Teimori, M. Ebrahimi and A. Gholamhoseini. SMF 33337, 1, 48.86 mm SL; Iran, Lorestan prov., Hadi River between Zagheh and Polehoru, 33°31.138'N, 48°46.340'E. 04 March 2008. N. Alwan, K. Borkenhagen, M. Ghanbari Fardi and A. Kazemi. FSJF 2213, 11, 107.92–143.94 mm SL; Iran, Chaharmahal and Bakhtiari Prov., Sandgan River (Sandgan stream) at Sandgan, 31°15.692'N, 51°17.150'E. 19 April 2007, A. Abdoli and J. Freyhof. FSJF 2233, 2, 156.22–162.23 mm SL; Iran, Kohgiluyeh and Boyer Ahmad prov., Beshar River, 20 km northeast of Yasuj, 30°44.152'N, 51°29.522'E. 19 April 2007. A. Abdoli and J. Freyhof. SMF 30865, 1, 26.94 mm SL; Iran, Kohgiluyeh and Boyer Ahmad prov., Beshar River at Tang-e Sorkh, 30°27.680'N, 51°44.907'E. 28 November 2007, K. Borkenhagen, H. R. Esmaeili and F. Wicker (in 96% alcohol). SMF 30871, 1, 28.34 mm SL; Iran, Kohgiluyeh and Boyer Ahmad prov., Beshar River at Tang-e Sorkh, 30°27.680'N, 51°44.907'E. 28 November 2007. K. Borkenhagen, H. R. Esmaeili and F. Wicker (in 96% alcohol). SMF 33316, 7, 35.22–166.87 mm SL; Iran, Kohgiluyeh and Boyer Ahmad prov., Beshar River at Tang-e Sorkh, 30°27.680'N, 51°44.907'E. 28 November 2007, K. Borkenhagen, H. R. Esmaeili and F. Wicker. SMF 30872, 1, 29.70 mm SL; Iran, Fars prov., Sepidan, Tang-e Tizab, 30°23.470'N, 51°46.710'E, 28 November 2007, K. Borkenhagen, H. R. Esmaeili and F. Wicker (in 96% alcohol).

##### *Capoeta coadi* specimens used for molecular genetic analysis.

ZM-CBSU M1275,1, Iran, Kohgiluyeh and Boyer Ahmad prov., Beshar River at Dehno village, Karun River drainage, 30°38'55"N, 51°37'05"E. 16 January 2014, H.R. Esmaeili, G. Sayyadzadeh, H.R. Mehraban, M. Razbani. GenBank accession number: (COI: KU564296); ZM-CBSU M1447, 2, GenBank accession number: (COI: KU564297, KU564298; cytb: KU564303, KU564304) ZM-CBSU M1458, 2); Iran, Kohgiluyeh and Boyer Ahmad prov., Beshar River at Tale Gah village, Karun River drainage, 30°47'27"N, 51°25'13"E. 14 December 2013. G. Sayyadzadeh, A. Khajehpanah, R. Khaefi. GenBank accession number: (COI: KU564294, KU564295; *cytb*: KU564305, KU564306).

##### Diagnosis.

*Capoeta coadi* sp. n. is distinguished from all other species of *Capoeta* by the following combination of characters: last unbranched dorsal-fin ray weakly to moderately ossified and serrated in 1/3–2/3 of its length; scales small, 70–84 total lateral line scales (84 in holotype), 12–17 scales between dorsal-fin origin and lateral line (16 in holotype), 9–11 scales between anal-fin origin and lateral line (11 in holotype), 26–32 encircling least circumference of caudal peduncle (31 in holotype); total gill rakers 14–18 (17 in holotype), 10–13 gill rakers on lower limb of first gill arch (12 in holotype); 45–47 total vertebrae; one posterior pair of barbels; length of the longest dorsal-fin ray 14.92–21.58% SL (18.90 in holotype); head length 22.87–26.33% SL (23.76 in holotype); mouth width 7.48–9.77% SL (8.65 in holotype); bright golden-greenish or silvery body coloration in life.

##### Description.

**Figure 1.**
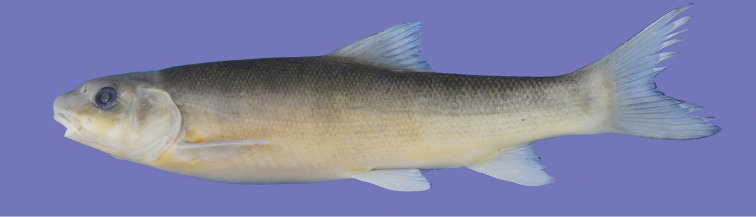
*Capoeta coadi* sp. n., ZM-CBSU Z190, holotype, 157 mm SL; Iran: Kohgiluyeh and Boyer Ahmad, Beshar River, Karun River drainage.

**Figure 2.**
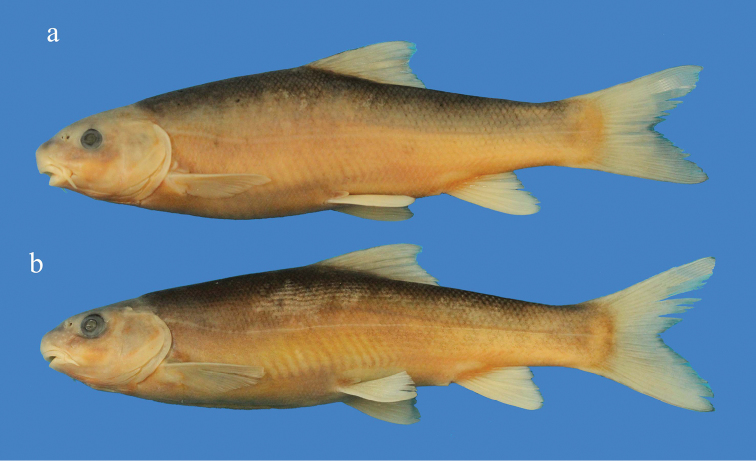
*Capoeta coadi* sp. n., paratypes: **a** ZM-CBSU Z191; 157 mm SL **b** ZM-CBSU Z192, 148 mm SL; Iran: Kohgiluyeh and Boyer Ahmad, Beshar River, Karun River drainage.

**Figure 3.**
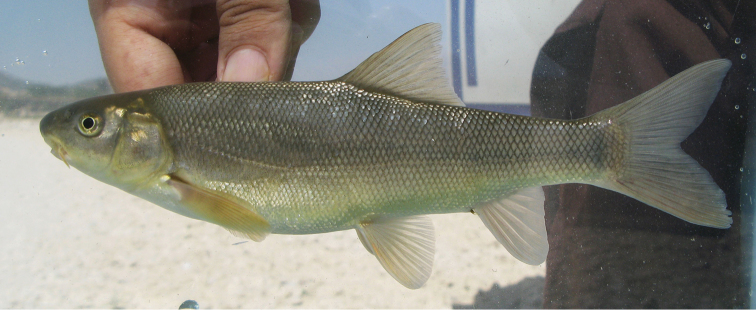
Live specimen of *Capoeta coadi* sp. n, Iran: Kohgiluyeh and Boyer Ahmad, Beshar River, Karun River drainage.

General body shape and appearance are shown in Figs 1–3, morphometric data in Table 1 and meristic data are summarized in Tables 2–9. Body elongate and cylindrical; predorsal body profile smoothly convex with no marked discontinuity between head and body except when a nuchal hump is present in few specimens; greatest body depth at level of dorsal-fin origin; snout rounded (in 20 specimens) or pointed (in 14 specimens) and not size dependent; mouth inferior; lips slightly fleshy, especially at the mouth corners; lower lip covered with a sharp-edged horny sheath, its anterior margin straight in adult specimens and rounded to almost crescent-shaped in juveniles, with a considerable degree of individual variation.

**Table 1.** Morphometric data of *Capoeta coadi* sp. n. (holotype ZM-CBSU Z190, and 33 paratypes), *Capoeta buhsei* and *Capoeta saadii*.

	Holotype	Paratypes (n=33)			*Capoeta buhsei* (n=27)			*Capoeta saadii* (n=20)		
		Range	Mean	SD	Range	Mean	SD	Range	Mean	SD
Standard length (mm)	157.64	67.23–157.64	110.67		74.30–149.30	112.56		51.31–231	109.30	
**In percent of standard length**										
Head length	23.76	22.87–26.33	24.5	0.80	21.47–25.98	23.56	0.98	24.28–29.62	26.84	1.32
Body depth at dorsal-fin origin	21.82	21.33–25.04	23.15	0.98	19.78–24.55	21.82	1.19	19.58–27.78	23.32	2.11
Predorsal length	49.07	47.75–53.43	50.23	1.31	48.85–55.05	51.59	1.34	44.33–55.12	51.93	2.51
Postdorsal length	54.13	54.13–63.19	57.53	1.88	48.20–60.13	55.24	2.78	50.79–59.64	55.06	2.60
Preanal length	72.45	70.22–76.14	72.8	1.36	71.34–76.34	74.01	1.28	69.37–78.38	75.61	2.07
Prepelvic length	53.74	50.22–55.90	52.84	1.31	50.17–56.64	53.58	1.49	51.23–61.21	56.22	2.36
Distance between pectoral and pelvic-fin origins	32.42	27.81–32.42	30.19	1.13	29.07–33.64	31.30	1.04	25.55–32.66	30.87	2.15
Distance between pelvic and anal-fin origins	21.48	19.31–23.17	21.12	0.88	19.90–23.65	21.60	0.91	18.32–23.41	20.83	1.56
Depth of caudal peduncle	10.37	10.03–11.61	10.65	0.37	8.58–10.84	10.05	0.50	8.98–11.15	10.43	0.60
Length of caudal peduncle	20.73	17.16–22. 35	19.85	1.14	18.64–22.01	19.81	0.91	15.19–20.11	17.67	1.30
Dorsal-fin base length	12.71	12.27–16.17	14.41	0.89	11.75–15.28	13.51	0.80	10.46–14.39	12.98	1.11
Anal-fin base length	6.78	6.38–8.85	7.39	0.55	6.96–8.80	7.88	0.58	6.24–8.27	7.17	0.60
Pectoral-fin length	17.32	16.68–20.46	18.43	0.89	16.39–20.96	18.38	0.99	16.15–19.16	17.86	1.01
Pelvic-fin length	15.05	14.24–16.96	15.61	0.68	13.85–18.08	15.61	1.08	13.58–16.23	15.08	0.82
Length of the longest dorsal fin ray	18.90	14.92–21.58	19.57	1.27	16.42–21.22	18.78	1.06	16.35–21.53	19.03	1.47
Mouth width	8.65	7.48–9.77	8.63	0.51	6.49–8.89	7.87	0.57	6.51–9.38	8.1	0.73
**In percent of head length**										
Head depth at eye	56.88	49.05–61.87	54.21	2.73	48.01–56.63	67.01	2.26	49.17–57.97	65.47	3.96
Snout length	38.32	31.60–47.70	38.08	2.60	32.69–38.89	35.55	1.67	32.65–40.61	36.18	2.41
Postorbital distance	48.83	33.82–51.84	48.01	3.01	47.66–56.59	51.57	1.89	46.58–54.84	51.05	2.28
Interorbital width	40.04	34.62–42.81	38.19	1.98	33.88–41.49	37.15	1.93	30.90–40.16	35.57	2.70
Eye diameter	15.97	15.07–23.57	18.52	2.36	13.91–24.44	17.36	2.11	11.95–26.18	18.23	3.43
Maximum head width	60.53	51.75–66.89	59.60	3.99	57.83–69.68	62.76	3.04	47.38–59.00	54.62	3.39
Barbel length	15.14	13.30–20.20	16.25	1.66	15.66–24.60	19.71	2.27	13.34–24.64	18.11	2.73

**Figure 4.**
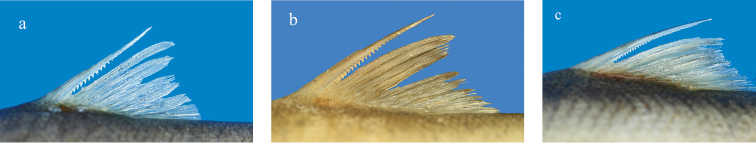
Dorsal fins of *Capoeta coadi* sp. n. **a** ZM-CBSU J 444; 73 mm SL **b** ZM-CBSU Z195; 104 mm SL **c** ZM-CBSU Z192; 148 mm SL; Iran: Kohgiluyeh and Boyer Ahmad, Beshar River, Karun River drainage, to show size-dependent variability of the last simple dorsal-fin ray serration.

Dorsal-fin origin anterior to pelvic-fin origin, its outer margin usually straight to concave with 3–5 unbranched and 8–9 branched rays (3 and 8 in holotype, respectively); last unbranched dorsal-fin ray weakly to moderately ossified, flexible and soft at the tip, serrated in 1/2–2/3 of its length (Fig. 4); pectoral fins not extending to pelvic-fin base; their outer margins usually slightly convex with 16–22 rays in total (19 in holotype) (Table 2); pelvic fins not extending to anal fin base, their outer margin straight or slightly convex and blunt with 7–11 rays in total (8 in holotype) (Table 2); pelvic axillary scale present; anal fin with 3 unbranched and 5 branched rays, outer margin straight or slightly convex; caudal fin forked with 16–19 branched rays (17 in holotype) (Table 3), its tip pointed and its upper lobe often longer than lower one.

**Table 2.** Number of pectoral and pelvic fin rays in examined *Capoeta* species.

	Pectoral fin rays										Pelvic fin rays					
	13	14	15	16	17	18	19	20	21	22		7	8	9	10	11
*Capoeta buhsei*			2	10	4	6						2	14	10		
*Capoeta coadi*				6	10	8	11	7	1	3		1	14	16	12	8
*Capoeta mandica*	1	7	2	1								9	2			
*Capoeta saadii*					3	12	4	1					9	10	1	
*Capoeta trutta*		2	8	17	8	5							22	17	1	

**Table 3.** Number of branched caudal fin rays in examined *Capoeta* species.

Branched caudal fin rays	15	16	17	18	19	20
*Capoeta buhsei*		3	21	3		
*Capoeta saadii*		1	29	2	1	
*Capoeta mandica*		2	8	1		
*Capoeta trutta*	1	9	16	11	3	

Scales small, total lateral-line scales 70–84; 12–17 scales between dorsal-fin origin and lateral line (Table 4); 9–11 scales between anal-fin origin and lateral line (Table 4); 26–32 circum-peduncle scales (Table 5); ventral midline and pectoral region covered with deeply embedded scales of reduced size; gill rakers slightly hooked, total gill rakers 14–18 (10–13 gill rakers on lower limb) of first gill arch (Table 8–9); 45–47 total vertebrae; usually one posterior pair of barbels present (very rarely two, 1 out of 51 individual); pharyngeal teeth arranged in 3 rows in the following manner: 2.3.5–5.3.2 and very similar in shape to those of *Capoeta damascina*; teeth in the main row spatulate or spoon-shaped and crowns flat, narrow and curved.

**Table 4.** Number of scales above (between dorsal-fin origin and lateral line) and below (between dorsal-fin origin and lateral line) lateral line in examined *Capoeta* species.

	Above lateral line												Below lateral line								
	6	7	8	9	10	11	12	13	14	15	16	17	5	6	7	8	9	10	11	12	13
*Capoeta buhsei*								3	6	4	12						3	13	7	3	1
*Capoeta coadi*							1	9	9	15	15	1					11	20	18		
*Capoeta mandica*							1	10								4	5	2			
*Capoeta saadii*				1	2	1	8	7	1					2	4	3	9	2			
*Capoeta trutta*			2	1	1	7	16	7	3	3						7	19	8	6		

**Table 5.** Number of circum-pendicular scales in examined *Capoeta* species.

	19	21	22	23	24	25	26	27	28	29	30	31	32	33
*Capoeta buhsei*							1	3	2	5	5	3	4	3
*Capoeta coadi*							3	11	7	14	4	7	5	
*Capoeta fusca*	1	6	2	4	1	1								
*Capoeta mandica*								5	1	3	1			1

**Coloration.** Live specimens. Dorsum and sides bright golden-green or silvery, darker dorsally and lighter below the lateral line; dorsal head bright golden-green or light pink-brown; dorsal, anal and caudal fins beige to light brown with light pink to red tinge; pectoral and pelvic-fins beige to light brown or golden with brown tinge on the first few rays (Fig. 3); few large black blotches present on the body of some specimens whereas small diffuse black spots are present only on the body of some juveniles (above the lateral line).

##### Preserved specimens.

Dorsum, head and sides grey or brownish-grey dorsally and beige or yellow ventrally; dorsal and caudal fins dusky grey; pectoral, pelvic and anal fins white or beige with or without grey tinge; blotches and spots well discernible (Figs 1–2).

##### Sexual dimorphism.

Breeding tubercles present in both sexes, being bigger and more pronounced in males. Tubercles present on the sides of the snout but may also cover the entire body surface, on and above the lateral line with one or two tubercles per scale but not on each scale, below the lateral line especially in the area above the anal fin and on the branched anal-fin rays; tip of anal fin reaching to or beyond the vertical of the caudal-fin base in females and to about 2/3 of the caudal peduncle in males.

##### Habitat and distribution.

**Figure 5.**
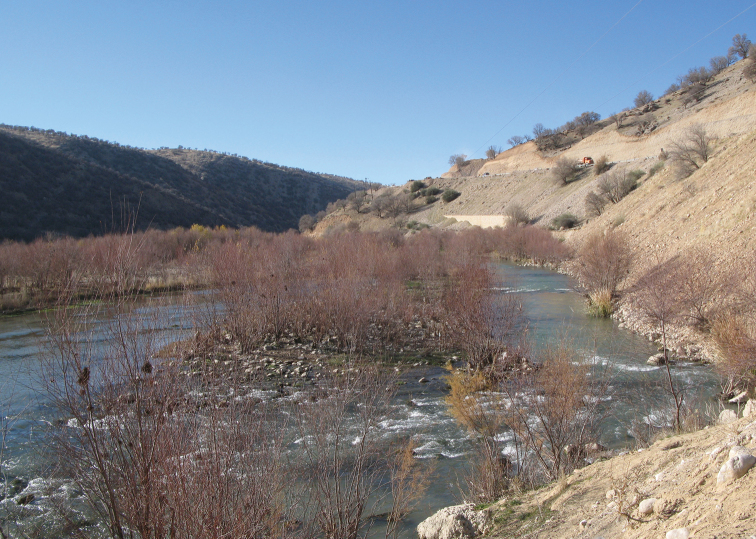
Beshar River at Taleh Gah village, Karun River drainage, type locality of *Capoeta coadi*.

*Capoeta coadi* sp. n. occurs in medium-fast flowing rivers with usually gravel substrates and clear waters (Fig. 5). At the Beshar River sampling site, the river is about 25 m wide, with substrate consisting of coarse gravel and boulders, and fast-flowing and semi-transparent waters. The physicochemical parameters at the spot were: dissolved oxygen, 9.89 mg/L; total dissolved solids, 190.2 mg/L; salinity, 0.19‰; conductivity, 395 µs/cm; pH: 8.5 and water temperature 23.4 °C. It is known only from the Karun River drainage, a system that constitutes the southeastern part of the Tigris-Euphrates River system.

##### Etymology.

The new species is named after Brian W. Coad, a well-known ichthyologist for his valuable contribution to the knowledge of freshwater fishes of Iran.

##### Comparative remarks.

The presence of one pair of barbels in *Capoeta coadi* sets the species apart from *Capoeta antalyensis*, *Capoeta baliki*, *Capoeta banarescui*, *Capoeta tinca*, and *Capoeta heratensis*, all of which have two pairs of barbels based on data from Turan et al. (2006a) and this study. The new species is further distinguished from *Capoeta antalyensis* by the presence of serrae on the last unbranched dorsal-fin ray (vs. absence) (Fig. 4), and by number of scales between dorsal-fin origin and lateral line (12–17 vs. 10–12 in *Capoeta antalyensis*) (Table 4), between anal-fin origin and lateral line (9–11 vs. 7), and by total number of the lateral-line scales (70–84 vs. 51–57) (Table 7). *Capoeta coadi* is distinguished from *Capoeta banarescui* by number of scales between anal-fin origin and lateral line (9–11 vs. 8–9) (Table 4). Data for *Capoeta antalyensis* and *Capoeta banarescui* are from Turan et al. (2006a).

*Capoeta coadi* is distinguished from *Capoeta mandica*, *Capoeta erhani*, and *Capoeta trutta* by having 10–13 gill rakers on the lower limb of the first gill arch (vs. 17–24 in *Capoeta mandica*, 20–22 in *Capoeta erhani* and 18–25 in *Capoeta trutta* [data from Krupp 1985, Turan et al. 2008, Table 8]). The total number of gill rakers in *Capoeta coadi* specimens is 14–18 that is lower than in *Capoeta mandica* (23–27), *Capoeta barroisi* (28–30), *Capoeta turani* (25–30) and *Capoeta trutta* (21–31) [data from Turan et al. (2006b), Özuluğ and Freyhof (2008), and this study] Table 9. *Capoeta coadi* is further distinguished from *Capoeta mandica* by having fewer pectoral fin rays (16–22 vs. 13–16) (Table 2). *Capoeta coadi* is distinguished from *Capoeta bergamae*, *Capoeta capoeta* and *Capoeta sieboldii* by number of scales between dorsal-fin origin and lateral line (12–17 in *Capoeta coadi* vs. 8–10 in *Capoeta capoeta* and 9–11 in *Capoeta sieboldii*); number of scales between anal-fin origin and lateral line (9–11 in *Capoeta coadi* vs. 7–9 in *Capoeta bergamae*, 6–10 in *Capoeta capoeta* and 8–10 in *Capoeta sieboldii*); total lateral line scales (70–84 in *Capoeta coadi* vs. 48–66 in *Capoeta capoeta* and 52–60 in *Capoeta sieboldii*) [data from Banarescu 1999, Turan et al. 2006b, Tables 4, 7]. In addition to the presence of serrae on the unbranched dorsal-fin ray, *Capoeta coadi* is set apart from *Capoeta caelestis* by the number of scales between the dorsal-fin origin and lateral line (12–17 in *Capoeta coadi* vs. 10–13.5 in *Capoeta caelestis*); scales between anal-fin origin and lateral line (9–11 in *Capoeta coadi* vs. 7–8 in *Capoeta caelestis*); circum-peduncular scales (26–32 in *Capoeta coadi* vs. 23–24 in *Capoeta caelestis*) (Tables 4–5) and probably vertebral counts (45–47 in *Capoeta coadi* vs. 44 in *Capoeta caelestis*) [data from Schöter et al. 2009].

**Table 6.** Number of caudal-peduncle scales in examined *Capoeta* species.

	10	11	12	13	14	15	16	17	18	19	20	21	22
*Capoeta buhsei*								6	3	7	9	1	1
*Capoeta coadi*					1	1	2	8	15	2	3	1	
*Capoeta mandica*					4	2	4		1				
*Capoeta saadii*					2	1	10	6	1				
*Capoeta trutta*		1	1		2	3	5	14	8	1	4		1

**Table 7.** Number of lateral-line scales in examined *Capoeta* species.

	58	59	61	62	63	64	65	66	67	68	69	70	71	72	73	74	75	76	77	78	79	80	81	82	83	84	85	87	89
*Capoeta buhsei*														1		3		1	2	1	2		4	3	1	2	4	1	1
*Capoeta coadi*												2	1	2	6	1	4	4	1	5	3	5	6	5	1	1			
*Capoeta mandica*	1		1	2	1		2	1	2	1																			
*Capoeta saadii*			1				2	1	2	2	1	2	3	1				1	2	2									
*Capoeta trutta*			1			2		1	3	1	5	4	2	2	2		5	3	2	4		2				1			

**Table 8.** Gill rakers on the lower limb of the first gill arch in studied *Capoeta* species.

GR	8	9	10	11	12	13	17	18	19	20	22	24
*Capoeta buhsei*	2	19	6									
*Capoeta coadi*			1	9	19	20						
*Capoeta mandica*							1	2	3	1	3	1

**Table 9.** Number of total gill rakers on the first gill arch in examined *Capoeta* species.

	12	13	14	15	16	17	18	21	22	23	24	25	26	27	28	31
*Capoeta buhsei*	10	13	6													
*Capoeta coadi*			1	7	14	6	5									
*Capoeta mandica*										2	2	2	1	4		
*Capoeta saadii*	1	9	6	1	2	1										
*Capoeta trutta*								1	1	9	11	7	5	4	1	1

It is distinguished from *Capoeta damascina* by having 11–13, modally 13, gill rakers on the lower limb of the first gill arch (vs. 12–18, modally 14–15) (Alwan 2011, Table 8). *Capoeta coadi* is clearly distinguished from *Capoeta ekmekciae* by number of scales between dorsal-fin origin and lateral line (12–17 in *Capoeta coadi* vs. 9–10 in *Capoeta ekmekciae*); number of scales between anal-fin origin and lateral line (9–11 in *Capoeta coadi* vs. 6–7 in *Capoeta ekmekciae*) (Table 4); number of lateral line scales (70–84 in *Capoeta coadi* vs. 55–61 in *Capoeta ekmekciae* [data from Turan et al. 2006b; Alwan 2011].

*Capoeta coadi* is distinguished from *Capoeta kosswigi* by total number of gill rakers (Table 9): 14–18 in *Capoeta coadi* vs. 19–28 in *Capoeta kosswigi* (see Karaman 1969; Turan et al. 2006b; Turan 2008).

*Capoeta coadi* is distinguished from *Capoeta mauricii* and *Capoeta pestai* by having a weaker, thinner and less ossified last unbranched dorsal-fin ray in juveniles and adults and fewer scales between dorsal-fin origin and lateral line (12–17 in *Capoeta coadi* vs. 18–22 in *Capoeta mauricii* and 16–19 in *Capoeta pestai* [data from Özuluğ and Freyhof 2008, Küçük et al. 2009]). It is further distinguished from *Capoeta pestai* by the absence of spots on the body except in juveniles (vs. presence of many on the body [see Özuluğ and Freyhof 2008, Küçük et al. 2009]). *Capoeta coadi* is distinguished from *Capoeta umbla* by total number of lateral line scales (70–84 vs. 86–104), number of scales between dorsal-fin origin and lateral line (12–17 vs. 18–24), number of scales between anal-fin origin and lateral line (9–11 vs. 11.5–15.5), and circum-pendicular scales (26–32 vs. 32–39) (see Alwan 2011, Tables 4–7).

Compared to other Iranian species of *Capoeta*, *Capoeta coadi* has more scales and fewer gill rakers than *Capoeta aculeata* (number of scales between dorsal-fin origin and lateral line: 12–17 vs. 6–10; number of scales between anal-fin origin and lateral line: 9–11 vs. 5–8; circum-peduncular scales: 26–32 vs. 13–23; total number of lateral line scales: 70–84 vs. 36–52; caudal peduncle scales: 14–18 vs. 10–12; gill rakers on the lower limb of the first gill arch: 10–13 vs. 15–18 [data from Coad and Krupp 1994] and this study (Tables 4–9)). *Capoeta coadi* is distinguished from *Capoeta fusca* by more total vertebrae (45–47 vs. 44), and more total lateral-line scales (70–84 vs. 40–62) (see Coad 2008, Johari et al. 2009).

**Figure 6.**
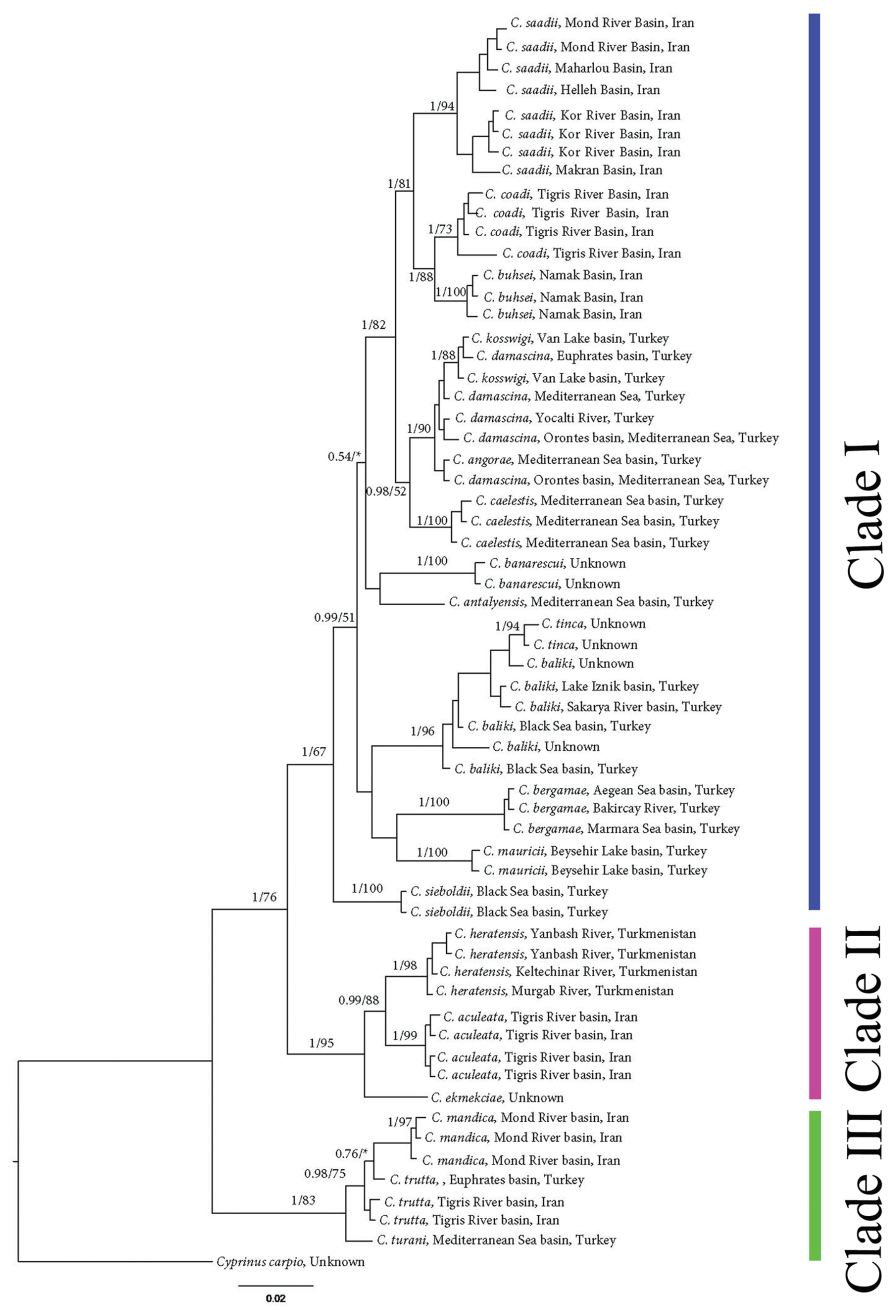
Bayesian tree inferred from *cyt b*. Numbers left of the slash, indicate the posterior probabilities of the Bayesian analysis, using MrBayes, while numbers right of the slash are the bootstrap support for 10,000 replicates in the Maximum Likelihood tree, using RaxML. Asterisks (*) indicate less than 50% Maximum Likelihood support for the node.

**Figure 7.**
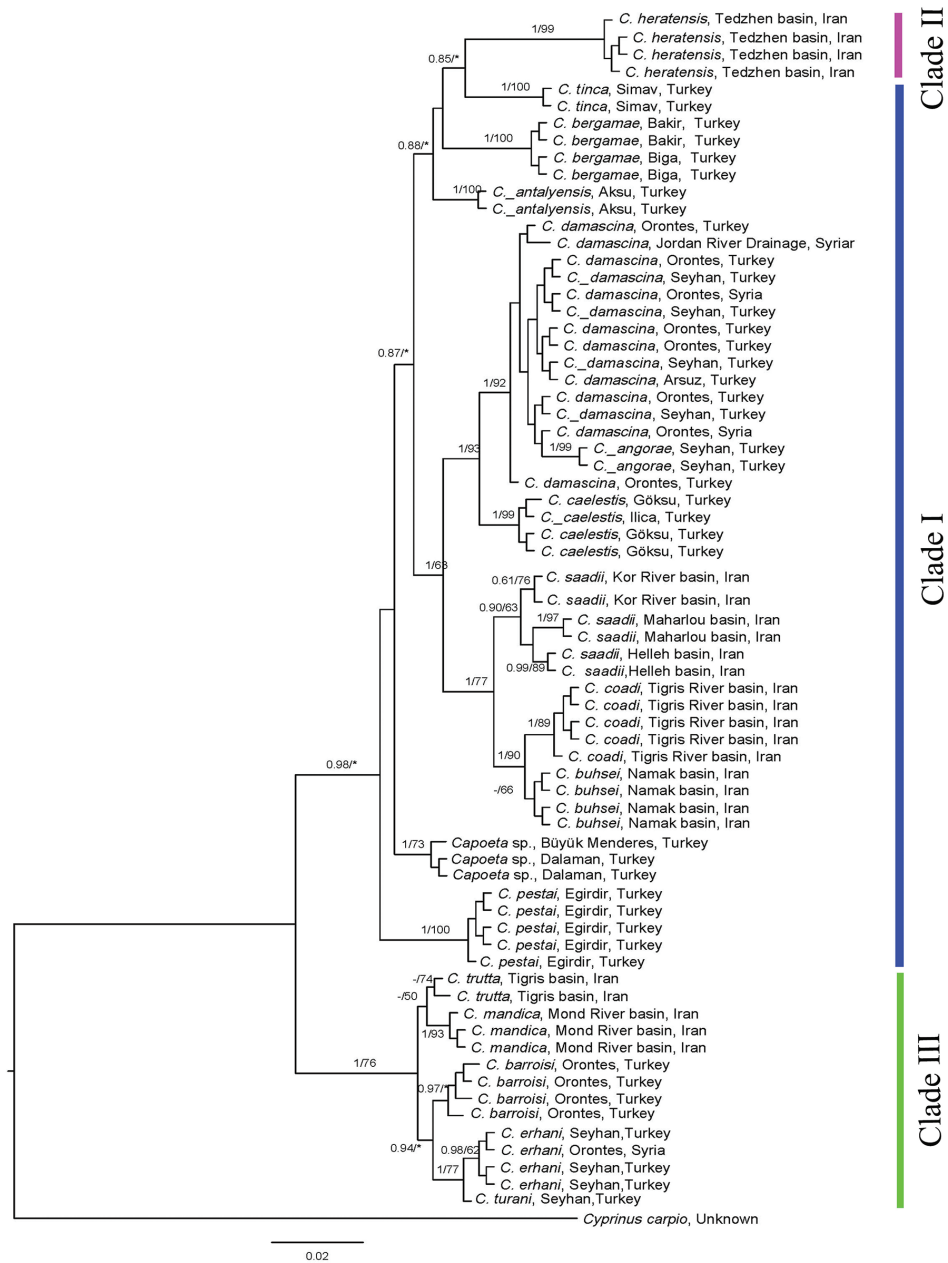
Bayesian tree inferred from COI. Numbers left of the slash, indicate the posterior probabilities of the Bayesian analysis, using MrBayes, while numbers right of the slash are the bootstrap support for 10,000 replicates in the Maximum Likelihood tree, using RaxML. Asterisks (*) indicate less than 50% Maximum Likelihood support and (-) indicates less than 0.50 Bayesian posterior probabilities for the node.

*Capoeta coadi* differs from its sister species (see Figs 6–7), *Capoeta buhsei* in having more gill rakers on lower limb of first gill arch (10–13 vs. 8–10), more gill rakers on the whole first gill arch (14–18 vs. 12–14, see Tables 8–9) and by depth of caudal peduncle in percent of standard length (10.03–11.61 vs. 8.58–10.84). *Capoeta coadi* is distinguished from another closely related species, *Capoeta saadii* by having more scales below the lateral line (9–11 vs. 6–10, modally 9) (Table 4) and more circum-pendicular scales (26–32 vs. 23–28, modally 25–26) [data from Alwan (2011)].

### Molecular phylogenetic assessments

We generated COI barcode and *cyt b* sequences for a total of 76 and 61 *Capoeta* specimens, respectively (Tables 10–11). Two phylogenetic approaches including Maximum Likelihood and Bayesian analyses for species of *Capoeta* are given in Figs 6–7. Tables 12–13 provide the diagnostic nucleotide substitutions found in the mtDNA COI barcode region and *cyt b*, respectively.

**Table 10.** List of species used for molecular analysis for *cyt b* (*present study, the ones without * are obtained from GenBank). *Cyprinus carpio* was considered as outgroup.

Species	Accession Number	Locality
*Capoeta aculeata*	JF798267	Stream Sangan, Karun River basin, Tigris basin, Iran
*Capoeta aculeata*	JF798264	Sevah River, Kor basin, Iran
*Capoeta aculeata*	JF798266	Beshar River, Karun basin, Tigris basin, Iran
*Capoeta aculeata*	JF798265	Sevah River, Kor basin, Iran
*Capoeta angorae*	JF798268	Pozanti River, Mediterranean Sea basin, Turkey
*Capoeta antalyensis*	JF798269	Boga Cayi River, Mediterranean Sea basin, Turkey
*Capoeta baliki*	JF798272	Kizilirmak River, Black Sea basin, Turkey
*Capoeta baliki*	JF798273	Biggest tributary of Kurtbog˘azi dam lake, Sakarya River basin, Turkey
*Capoeta baliki*	JF798275	Stream Cakirca, Lake Iznik basin, Turkey
*Capoeta baliki*	GQ424019	Unknown
*Capoeta baliki*	GQ424020	Unknown
*Capoeta baliki*	JF798271	Kizilirmak River, Black Sea basin, Turkey
*Capoeta banarescui*	GQ423987	Unknown
*Capoeta banarescui*	GQ423988	Unknown
*Capoeta bergamae*	JF798282	Bakacak stream, Marmara Sea basin, Turkey
*Capoeta bergamae*	JF798280	Bakircay River, Turkey
*Capoeta bergamae*	JF798281	Stream Guzelhisar, Aegean Sea basin, Turkey
*Capoeta buhsei*	JF798283	Taghra Rud stream, Namak Lake basin, Iran
Capoeta buhsei*	KU312369	Kordan River, Namak Lake basin, Karaj, Iran
Capoeta buhsei*	KU312370	Kordan River, Namak Lake basin, Karaj, Iran
*Capoeta caelestis*	JF798336	Ilica stream, Gulf of Antalya, Mediterranean Sea basin,Turkey
*Capoeta caelestis*	JF798286	Goksu River, Mediterranean Sea basin, Turkey
*Capoeta caelestis*	JF798287	Kargi Cayi River, Mediterranean Sea basin, Turkey
Capoeta coadi*	KU564303	Beshar River, Tigris River basin, Iran
Capoeta coadi*	KU564304	Beshar River, Tigris River basin, Iran
Capoeta coadi*	KU564305	Beshar River, Tigris River basin, Iran
Capoeta coadi*	KU564306	Beshar River, Tigris River basin, Iran
*Capoeta damascina*	JF798309	Karadut River, Euphrates basin, Turkey
*Capoeta damascina*	JF798303	Stream Arsuz, Iskenderun Gulf basin, Mediterranean Sea, Turkey
*Capoeta damascina*	JF798308	Yocalti River, Turkey
*Capoeta damascina*	JF798306	Spring Incesu, Orontes basin, Mediterranean Sea, Turkey
*Capoeta damascina*	JF798307	Yocalti River, Mediterranean Sea basin, Turkey
*Capoeta ekmekciae*	GQ424027	Unknown
*Capoeta heratensis*	JF798319	Keltechinar River, Turkmenistan
*Capoeta heratensis*	JF798318	Yanbash River, Turkmenistan
*Capoeta heratensis*	JF798317	Yanbash River, Turkmenistan
*Capoeta heratensis*	JF798316	Murgab River, Turkmenistan
*Capoeta kosswigi*	JF798322	Deli Cayi River, Van Lake basin, Turkey
*Capoeta kosswigi*	JF798323	Deli Cayi River, Van Lake basin, Turkey
*Capoeta mandica* *	KU564307	Ghare Aghaj River, Mond River basin, Khaneh Zanian , Iran
*Capoeta mandica* *	KU564308	Ghare Aghaj River, Mond River basin, Khaneh Zanian , Iran
*Capoeta mandica* *	KU312375	Ghare Aghaj River, Mond River basin, Khaneh Zanian , Iran
*Capoeta mauricii*	JF798325	Eflatum spring, Beysehir Lake basin, Turkey
*Capoeta mauricii*	JF798324	Sarioz stream, Beysehir Lake basin, Turkey
Capoeta saadii*	KU564309	Ghare Aghaj River, Mond River basin, Firuzabad, Iran
Capoeta saadii*	KU564310	Ghare Aghaj River, Mond River basin, Firuzabad , Iran
Capoeta saadii*	KU564311	Saadii Tomb Spring, Maharlu basin, Iran
Capoeta saadii*	KU312373	Helleh River, Helleh basin, KohmarSorkhi, Iran
Capoeta saadii*	KU564312	Kor River, Kor basin, Kamfirouz, Iran
Capoeta saadii*	KU564313	Kor River, Kor basin, Kamfirouz, Iran
*Capoeta saadii*	JF798326	Rodan River, Makran basin, Iran
*Capoeta saadii*	JF798327	Spring Golabii, 35 km north from Darab, Hormuz basin, Iran
*Capoeta sieboldii*	JF798329	Kizilirmak River, Black Sea basin, Turkey
*Capoeta sieboldii*	JF798330	Kelkit Cayi River, Black Sea basin, Turkey
*Capoeta tinca*	GQ424008	Unknown
*Capoeta tinca*	GQ424007	Unknown
*Capoeta trutta*	JF798334	Dez River, Karun River basin, Iran
*Capoeta trutta*	JF798333	Sultansuyu River, Euphrates basin, Turkey
*Capoeta trutta*	JF798332	Gelal River, Ab e Seymareh, Tigris River basin, Iran
*Capoeta turani*	JF798335	Çatkit River, Mediterranean Sea basin, Turkey
*Cyprinus carpio*	DQ868875	Unknown

**Table 11.** List of species used for molecular analysis for COI (*present study, the ones without * are obtained from GenBank). *Cyprinus carpio* was considered as outgroup.

Species	Accession num.	Locality
Capoeta heratensis*	KU564288	Gilas spring, Tedzen basin, Iran
Capoeta heratensis*	KU564289	Gilas spring, Tedzen basin, Iran
Capoeta heratensis*	KU564290	Gilas spring, Tedzen basin, Iran
Capoeta heratensis*	KU564291	Bezangan Lake, Tedzen basin, Iran
*Capoeta angorae*	KJ553074	Seyhan, Turkey
*Capoeta angorae*	KJ552868	Seyhan, Turkey
*Capoeta antalyensis*	KJ552850	Aksu, Turkey
*Capoeta antalyensis*	KJ553025	Aksu, Turkey
*Capoeta barroisi*	KJ553267	Orontes, Turkey
*Capoeta barroisi*	KJ553245	Orontes, Turkey
*Capoeta barroisi*	KJ552785	Orontes, Turkey
*Capoeta barroisi*	KJ552810	Orontes, Turkey
*Capoeta bergamae*	KJ553157	Bakir, Turkey
*Capoeta bergamae*	KJ552877	Bakir, Turkey
*Capoeta bergamae*	KJ553253	Biga, Turkey
*Capoeta bergamae*	KJ553081	Biga, Turkey
Capoeta buhsei*	KU312349	Kordan River, Namak Lake basin, Karaj, Iran
Capoeta buhsei*	KU312350	Kordan River, Namak Lake basin, Karaj, Iran
Capoeta buhsei*	KU564292	Roudbar River, Kavir basin,Iran
Capoeta buhsei*	KU564293	Roudbar River, Kavir basin,Iran
*Capoeta caelestis*	KJ552856	Göksu, Turkey
*Capoeta caelestis*	KJ553237	Ilica, Turkey
*Capoeta caelestis*	KJ553301	Göksu, Turkey
*Capoeta caelestis*	KJ553030	Göksu, Turkey
*Capoeta damascina*	KJ553080	Arsuz, Turkey
*Capoeta damascina*	KJ553043	Orontes, Turkey
*Capoeta damascina*	KJ552896	Orontes, Turkey
*Capoeta damascina*	KJ553272	Orontes, Turkey
*Capoeta damascina*	KJ552846	Orontes, Turkey
*Capoeta damascina*	KJ552874	Ceyhan, Turkey
*Capoeta damascina*	KJ552797	Orontes, Syria
*Capoeta damascina*	KJ553202	Orontes, Syria
*Capoeta damascina*	KJ553027	Ceyhan, Turkey
*Capoeta damascina*	KJ553194	Ceyhan, Turkey
*Capoeta damascina*	KJ552763	Ceyhan, Turkey
*Capoeta damascina*	KJ552939	Jordan River Drainage, Syria
*Capoeta damascina*	KJ553216	Orontes, Turkey
*Capoeta damascina*	KJ553089	Orontes, Turkey
*Capoeta erhani*	KJ552767	Ceyhan, Turkey
*Capoeta erhani*	KJ552087	Ceyhan, Turkey
*Capoeta erhani*	KJ552806	Ceyhan,Turkey
*Capoeta erhani*	KJ553067	Ceyhan,Turkey
Capoeta mandica*	KU564301	Ghare Aghaj River, Mond River basin, Khaneh Zanian, Iran
Capoeta mandica*	KU564302	Ghare Aghaj River, Mond River basin, Khaneh Zanian, Iran
Capoeta mandica*	KU312368	Ghare Aghaj River, Mond River basin, Khaneh Zanian, Iran
*Capoeta pestai*	KJ553304	Egirdir, Turkey
*Capoeta pestai*	KJ553138	Egirdir, Turkey
*Capoeta pestai*	KJ552113	Egirdir, Turkey
*Capoeta pestai*	KJ552841	Egirdir, Turkey
*Capoeta pestai*	KJ552818	Egirdir, Turkey
*Capoeta tinca*	KJ553229	Simav, Turkey
*Capoeta tinca*	KJ553168	Simav, Turkey
Capoeta trutta*	KU312352	Karkheh River, Tigris River basin, Seymareh, Iran
Capoeta trutta*	KU312351	Gavi River, Tigris River basin, Illam, Iran
*Capoeta turani*	KJ553224	Ceyhan Nehri, Turkey
Capoeta saadii*	KU312358	Saadii Tomb Spring, Maharlou basin, Iran
Capoeta saadii*	KU312395	Spring Pirbanoo, Maharlou basin, Iran
Capoeta saadii*	KU312360	Helleh River, Helleh basin, KohmarSorkhi, Iran
Capoeta saadii*	KU312361	Helleh River, Helleh basin, KohmarSorkhi, Iran
Capoeta saadii*	KU564299	Kor River, Kor basin, Kamfiruz, Iran
Capoeta saadii*	KU564300	Kor River, Kor basin, Kamfiruz, Iran
Capoeta saadii*	KU312359	Kor River, Kor basin, Kamfiruz, Iran
Capoeta coadi*	KU564294	Beshar River, Tigris River basin, Iran
Capoeta coadi*	KU564295	Beshar River, Tigris River basin, Iran
Capoeta coadi*	KU564296	Beshar River, Tigris River basin, Iran
Capoeta coadi*	KU564297	Beshar River, Tigris River basin, Iran
Capoeta coadi*	KU564298	Beshar River, Tigris River basin, Iran
*Capoeta* sp.	KJ552935	Dalaman, Turkey
*Capoeta* sp.	KJ553011	Büyük Menderes, Turkey
*Capoeta* sp.	KJ552882	Dalaman, Turkey
*Cyprinus carpio*	DQ868875	Unknown

**Table 12.** Diagnostic nucleotide substitutions found in mtDNA COI barcode region of *Capoeta* species. Nucleotide position relative to *Cyprinus carpio* complete mitochondrial genome.

Species	6545	6620	6626	6665	6683	6713	6749	6758	6761	6770	6818	6845	6875	6887	6905	6986	6995	7076	7088
*Capoeta buhsei*	C	A	A	T	G	G	T	C	G	G	G	A	A	C	C	G	A	G	T
*Capoeta caelestis*	T	G	C	T	G	A	G	T	G	A	A	C	G	T	T	G	G	G	C
*Capoeta coadi*	C	A	A	T	A	G	T	C	G	G	G	A	A	C	C	A	A	G	T
*Capoeta damscina*	T	A	G	T	G	A	G	T	A	A	G	C	A	T	T	G	G	A	C
*Capoeta saadii*	T	A	A	C	G	G	T	C	A	A	G	A	A	C	C	T	A	G	T

**Table 13.** Diagnostic nucleotide substitutions found in *cyt* b of *Capoeta* species. Nucleotide position relative to *Cyprinus carpio* complete mitochondrial genome.

Species	15430	15451	15457	15463	15472	15526	15550	15610	15670	15682	15760	15814	15925	16011	16027	16039	16045	16063
*Capoeta buhsei*	C	G	T	A	A	G	G	T	G	T	G	G	G	C	C	A	G	C
*Capoeta caelestis*	T	A	C	A	A	G	G	A	A	C	A	A	G	T	T	G	A	T
*Capoeta coadi*	T	G	C	A	G	G	A	C	G	T	G	G	G	C	C	A	G	C
*Capoeta damascina*	T	A	C	G	A	A	A	T	A	T	G	A	A	C	T	G	A	T
*Capoeta saadii*	T	G	C	G	A	A	A	T	A	T	G	A	A	C	T	G	A	T

For inter-specific differences, the greatest pairwise genetic divergence between *Capoeta coadi* and its congeners was found to be 6.5 by *Capoeta erhani* and lowest by *Capoeta buhsei* (0.4) for COI and greatest 9.7 by *Capoeta mandica* and lowest (1.5) by *Capoeta buhsei* for *cyt b* (Tables 14–15).

**Table 14.** Mean genetic distance for *cyt b* between *Capoeta* species.

	*Capoeta sieboldii*	*Capoeta caelestis*	*Capoeta mauricii*	*Capoeta bergamae*	*Capoeta baliki*	*Capoeta antalyensis*	*Capoeta tinca*	*Capoeta banarescui*	*Capoeta turani*	*Capoeta trutta*	*Capoeta buhsei*	*Capoeta coadi*	*Capoeta mandica*	*Capoeta saadii*
*Capoeta sieboldii*														
*Capoeta caelestis*	3.8													
*Capoeta mauricii*	5.1	4.6												
*Capoeta bergamae*	5.3	5.4	4.8											
*Capoeta baliki*	4.4	4.7	4.5	5.7										
*Capoeta antalyensis*	4.3	4.1	4.1	4.9	4.6									
*Capoeta tinca*	5.3	5.6	5.3	6.6	1.0	5.6								
*Capoeta banarescui*	5.7	4.9	4.9	6.0	4.9	4.4	4.9							
*Capoeta turani*	8.1	8.4	9.3	9.5	8.5	8.6	8.8	11.0						
*Capoeta trutta*	8.7	8.7	9.1	9.9	9.1	9.2	9.4	10.9	1.2					
*Capoeta buhsei*	4.3	2.6	4.1	5.6	4.3	4.0	5.1	4.7	9.3	9.5				
*Capoeta coadi*	4.2	2.6	4.5	6.0	5.0	4.3	5.8	5.4	9.4	9.6	1.5			
*Capoeta mandica*	8.5	8.8	9.0	9.9	8.7	9.5	9.2	11.4	1.5	1.1	9.6	9.7		
*Capoeta saadii*	4.8	3.3	4.8	4.6	4.7	4.8	5.4	5.8	8.5	8.9	2.8	2.7	9.1	
*Capoeta aculeata*	6.5	6.6	7.2	7.7	7.4	6.5	8.0	7.5	9.2	9.2	6.8	7.0	9.7	6.8

**Table 15.** Mean genetic distance for COI gene between *Capoeta* species.

	*Capoeta trutta*	*Capoeta heratensis*	*Capoeta buhsei*	*Capoeta coadi*	*Capoeta saadii*	*Capoeta pestai*	*Capoeta caelestis*	*Capoeta damascina*	*Capoeta barroisi*	*Capoeta bergamae*	*Capoeta tinca*	*Capoeta erhani*	*Capoeta angorae*	*Capoeta antalyensis*	*Capoeta mauricii*	*Capoeta mandica*
*Capoeta trutta*																
*Capoeta heratensis*	7.15															
*Capoeta buhsei*	6.02	5.10														
*Capoeta coadi*	6.01	5.23	0.44													
*Capoeta saadii*	6.22	5.16	1.12	1.42												
*Capoeta pestai*	5.82	5.07	3.83	3.60	3.82											
*Capoeta caelestis*	6.03	4.54	2.61	3.10	2.88	4.01										
*Capoeta damascina*	5.65	4.31	2.56	3.05	2.49	3.65	1.24									
*Capoeta barroisi*	0.57	6.72	5.99	6.52	6.17	6.29	5.93	5.52								
*Capoeta bergamae*	6.56	4.87	3.95	3.81	3.98	4.33	3.64	3.45	6.75							
*Capoeta tinca*	4.39	5.32	4.50	4.77	4.49	5.02	4.44	4.01	4.21	4.47						
*Capoeta erhani*	0.94	6.74	6.03	6.55	6.20	6.18	5.83	5.27	0.99	6.35	4.18					
*Capoeta angorae*	6.37	4.68	3.28	3.41	3.02	3.97	1.91	0.74	6.27	3.78	4.46	5.97				
*Capoeta antalyensis*	5.25	4.15	2.58	2.71	2.92	2.91	2.42	2.76	5.34	2.73	3.64	5.25	3.08			
*Capoeta mauricii*	5.82	5.07	3.83	3.60	3.82	0.00	4.01	3.65	6.29	4.33	5.02	6.18	3.97	2.91		
*Capoeta mandica*	0.42	7.31	6.22	6.34	6.39	5.99	6.20	5.83	0.73	6.54	4.58	1.18	6.54	5.43	5.99	
*Cyprinus carpio*	15.32	14.89	15.13	14.97	14.23	15.85	16.32	15.39	15.53	15.57	15.45	16.06	15.34	15.61	15.85	15.87

The two different phylogenetic analyses produced similar topologies. Both analyses produced a tree with 3 major clades (Figs 6–7). These included Clade I) *Capoeta antalyensis*, *Capoeta baliki*, *Capoeta banarescui*, *Capoeta bergamae*, *Capoeta buhsei*, *Capoeta caelestis*, *Capoeta coadi*, *Capoeta damascina* (*Capoeta angorae* is a synonym [Alwan 2011]), *Capoeta kosswigi*, *Capoeta mauricii*, *Capoeta pestai*, *Capoeta saadii*, *Capoeta sieboldii*, and *Capoeta tinca*, Clade II) *Capoeta aculeata*, *Capoeta ekmeckciae*, and *Capoeta heratensis*, and Clade III) *Capoeta barroisi*, *mandica*, *Capoeta trutta*, and *Capoeta turani*.

The Iranian members of the *Capoeta damascina* species complex, clustered together and formed the sister group to the other members in the complex. In these trees, samples of the *Capoeta coadi*, from Beshar River in Tigris River basin, form a well-supported monophyletic group, sister to *Capoeta buhsei* in clade I.

## Discussion

Based on morphological and molecular results, *Capoeta saadii* and *Capoeta coadi* are distinct species in the *Capoeta damascina* species complex group formerly known as *Capoeta damascina* in Iranian water bodies. Phylogenetic analyses recovered three main groups inside the genus *Capoeta*: the Mesopotamian group (*Capoeta trutta* group), the Anatolian-Iranian group (*Capoeta damascina* group) and the Aralo-Caspian group (*Capoeta capoeta* group) which is in agreement with Levin et al. (2012). The genus *Capoeta* is monophyletic (Levin et al. 2012). Based on the previous published data, the *Capoeta damascina* species complex group diverged from the *Capoeta capoeta* group about 9.1 MYA (95% CI: 6.4–10.9) in the Tortonian period (Levin et al. 2012). Iranian members of the *Capoeta damascina* group (*buhsei*, *saadii* and *coadi*) formed a clade sister to other *Capoeta damascina* species complex group members.

The populations of *Capoeta* from the Karun River drainage have long been considered as *Capoeta damascina* (Esmaeili et al. 2010). However, it has been proposed that *Capoeta damascina* might be restricted to the Damascus area in Syria. Most Iranian populations, referred to *Capoeta damascina*, including Karun River population have been considered as *Capoeta saadii* (Heckel, 1847) (Teimori et al. 2016). *Capoeta saadii* was originally described from Persepolis, Pulwar (Sivand) River, Kor River basin, Ruins, northeast of Shiraz, Iran. It was considered as a synonym of *Capoeta damascina* (Esmaeili et al. 2010) and as a valid species by Bianco and Bănărescu (1982), by Levin et al. (2012) and by Teimori et al. (2016). Based on morphological and molecular results presented here, *Capoeta saadii* is a valid species closely related to *Capoeta buhsei* (as proposed by Bianco and Bănărescu (1982) and to *Capoeta coadi* yet is diagnosed from these species and from *Capoeta damascina* (see Alwan 2011). *Capoeta saadii* is the least known species of the genus. It is not mentioned in the revision of the genus by Karaman (1969) who had no specimens available, but its position within the genus *Capoeta* and its close phylogenetic relationship to *Capoeta coadi* and *Capoeta buhsei* were demonstrated using many fresh specimens at our disposal, mostly from type localities.

## Comparative materials used in morphological and molecular phylogenetic analyses

### Morphological analyses

*Capoeta buhsei*: ZM-CBSU Z218-229, 12, 104-149 mm SL; Iran, Semnan prov., Kavir basin, Hableh Rud at Garmsar, 35°18'06"N, 52°24'57"E. 21 August 2011. H.R. Esmaeili, G. Sayyadzadeh, A. Gholamifard, R. Zamaniannejad. ZM-CBSU Z260-274, 15, 88–130 mm SL; Iran, Albourz prov., Kordan River at Karaj, 35°57'12"N, 56°50'18"E. 5 July 2014. M. Masoudi, R. Khaefi. H.R. Mehraban.

*Capoeta fusca*: ZM-CBSU Z197-211, 15, 50–78 mm SL; Iran, south Khorasan prov., Sharifabad Qanat at Birjand, 33°58'08"N, 59°17'03"E. 29 August 2011. H.R. Esmaeili, G. Sayyadzadeh, A. Gholamifard, R. Zamaniannejad.

*Capoeta mandica*: ZM-CBSU Z230-234, 5, 82-130 mm SL; Iran, Fars prov., Qareh Aghaj River at Khaneh Zanian, 29°41'13"N, 52°05'58"E. 30 May 2015. H. Zareian, A. Gholamhosseini, G. Sayyadzadeh. ZM-CBSU Z212-217, 6, 83-118 mm SL; Iran, Fars prov., Qareh Aghaj River at Kavar, 29°10'55"N, 52°41'32"E. 27 February 2015. G. Sayyadzadeh, M. Masoudi.

*Capoeta saadii*: ZM-CBSU Z136-146, 11, 78-121 mm SL; ZM-CBSU 2504, 1, 82 mm SL; ZM-CBSU 2508, 1, 69 mm SL; ZM-CBSU 2520-2521, 2, 51-62 mm SL; ZM-CBSU 2524-2528, 5, 113-231 mm SL; Iran, Fars prov., Ghadamgah spring, Doroodzan, 30°15'11"N, 54°25'32"E. 21 December 2003. H.R. Esmaeili, Biglari.

*Capoeta trutta*: ZM-CBSU E100-123, 24, 50-149 mm SL; Iran, Kermanshah prov., Gamasiab River, 34°23'31"N, 47°42'57"E. 27 September 2007. A. Teimori, A. Gholamhosseini, M. Ebrahimi, A. Gholamifard; ZM-CBSU C453-463, 11, 67-177 mm SL; ZM-CBSU C474-477, 4, 67–75 mm SL; ZM-CBSU C481, 76 mm SL; all from Iran, Khuzestan prov., Maroon River at Aghajari, 30°44'52"N, 49°54'59"E. 21 March 2008. H. Zareian.

### Molecular phylogenetic analyses

*Capoeta buhsei*; ZM-CBSU M1299-1300, 2, Iran, Albourz prov., Kordan River at Karaj, 35°57'12"N, 56°50'18"E. 5 July 2014. M. Masoudi, R. Khaefi. H.R. Mehraban. GenBank accession number: (COI: KU312349, KU312350; *cytb*: KU312369, KU312370); ZM-CBSU M1289-1290, 2, Iran: Semnan Prov., Kavir basin, Roudbar River at Mehdishahr, 35°37'56"N, 53°20'41"E. 30 August 2011. H.R. Esmaeili et al., GenBank accession number: (COI: KU564292, KU564293).

*Capoeta heratensis*; ZM-CBSU M813-815, 3, Iran, Razavi Khorasan prov., Gilas spring, 36°36'55"N, 59°20'17"E. 25 August 2011. H.R. Esmaeili et al. GenBank accession number: (COI: KU564288, KU564289, KU564290). ZM-CBSU M816, 1, Iran, Razavi Khorasan prov., Bezangan Lake, Tedzen basin. 36°17'03"N, 60°24'18"E. 25 August 2011. H.R. Esmaeili et al. GenBank accession number: (COI: KU564291).

*Capoeta mandica*: ZM-CBSU M1433-1435, 3, Iran, Fars prov., Qareh Aghaj River at Khaneh Zanian, 29°41'13"N, 52°05'58"E. 30 May 2015. H. Zareian, A. Gholamhosseini, G. Sayyadzadeh. GenBank accession number: (COI: KU564301, KU564302, KU312368; *cytb*: KU564307, KU564308, KU312375).

*Capoeta saadii*: ZM-CBSU M1426-1427, 2, Iran: Fars prov. Kor River, at Kamfirouz, 30°25'2"N, 52°8'59"E. H. Zareian. 24 October 2015. GenBank accession number: (COI: KU564299, KU564300; cytb: KU564312, KU564313). ZM-CBSU M1421, ZM-CBSU1422-1425, 3, Iran, Fars prov., Qareh Aghaj River at Firuzabad, 28°41'31"N, 52°27'43"E. 25 April 2015. H. Zareian. GenBank accession number: (*cytb*: KU564309, KU564310, KU564311). ZM-CBSU M157, 1, Iran, Fars prov., Shiraz, Saadii Tomb, Maharlou basin, 29°37.348'N, 52° 34.934'E. R. Khaefi, 2009. GenBank accession number: (COI: KU312358). ZM-CBSU M825, M831, 2, Iran, Fars prov., Helleh River, Helleh basin, KohmarhSorkhi, S. Mirgheiasi, S. Ghasemian. 29°23'39"N, 52°09'49"E. GenBank accession number: (COI: KU312361, KU312360; *cytb*: KU312373). ZM-CBSU M822, 1, Iran, Fars prov., Qareh Aghaj River at Firuzabad, 29°07'34"N, 52°51'24"E. GenBank accession number: (*cytb*: KU564310). FSJF DNA-18 Iran: Fars prov.: spring Pirbanoo about 10 km south of Shiraz, 29°31'08"N, 52°27'55"E GenBank accession number: (COI: KU312395). FSJF DNA-22; Iran: Fars prov.: River Kor about 73 km north of Shiraz, 30°11'37"N, 52°27'56"E. GenBank accession number: (COI: KU312359).

*Capoeta trutta*: ZM-CBSU M583, 1, Iran: Ilam prov.; Gavi River at Mehran, H.R. Esmaeili, 13 November 2012, 33°39'18"N, 47°02'14"E. GenBank accession number: COI: KU312351; ZM-CBSU M593, 1, Iran, Ilam prov.; Seymareh River, H.R. Esmaeili, 13 November 2012, 33°38'17"N, 47°01'30"E. GenBank accession number: COI: KU312352.

## Supplementary Material

XML Treatment for Capoeta coadi
